# Methods for training collaborative biostatisticians

**DOI:** 10.1017/cts.2020.518

**Published:** 2020-08-04

**Authors:** Gina-Maria Pomann, L. Ebony Boulware, Shari Messinger Cayetano, Manisha Desai, Felicity T. Enders, John A. Gallis, Jonathan Gelfond, Steven C. Grambow, Alexandra L. Hanlon, Angelean Hendrix, Pandurang Kulkarni, Jodi Lapidus, Hui-Jie Lee, Jonathan D. Mahnken, Julie P. McKeel, Rebecca Moen, Robert A. Oster, Sarah Peskoe, Greg Samsa, Thomas G. Stewart, Tracy Truong, Lisa Wruck, Samantha M. Thomas

**Affiliations:** 1 Department of Biostatistics and Bioinformatics, Duke University, Durham, NC, USA; 2 Division of General Internal Medicine, Department of Medicine, Duke University, Durham, NC, USA; 3 Division of Biostatistics, Department of Public Health Sciences, University of Miami, Miami, FL, USA; 4 Quantitative Sciences Unit, Department of Medicine, Stanford University, Stanford, CA, USA; 5 Department of Health Sciences Research, Mayo Clinic, Rochester, MN, USA; 6 Biostatistics Division, Department of Epidemiology & Biostatistics, University of Texas Health Science Center San Antonio, San Antonio, TX, USA; 7 Center for Biostatistics and Health Data Science, Department of Statistics, Virginia Tech, Roanoke, VA, USA; 8 Covance Inc., Princeton, NJ, USA; 9 Global Statistical Sciences, Eli Lilly and Company, Indianapolis, IN, USA; 10 School of Public Health, Oregon Health & Science University, Portland, OR, USA; 11 Department of Biostatistics & Data Science, University of Kansas Medical Center, Kansas City, KS, USA; 12 Duke Clinical and Translational Science Institute, Duke University, Durham, NC, USA; 13 Division of Preventive Medicine, Department of Medicine, University of Alabama at Birmingham, Birmingham, AL, USA; 14 Department of Biostatistics, Vanderbilt University Medical Center, Nashville, TN, USA; 15 Duke Clinical Research Institute, Duke University, Durham, NC, USA; 16 Duke Cancer Institute, Duke University, Durham, NC, USA

**Keywords:** Collaborative biostatistician, training strategy, quantitative collaboration, professional development, collaboration and communication

## Abstract

The emphasis on team science in clinical and translational research increases the importance of collaborative biostatisticians (CBs) in healthcare. Adequate training and development of CBs ensure appropriate conduct of robust and meaningful research and, therefore, should be considered as a high-priority focus for biostatistics groups. Comprehensive training enhances clinical and translational research by facilitating more productive and efficient collaborations. While many graduate programs in Biostatistics and Epidemiology include training in research collaboration, it is often limited in scope and duration. Therefore, additional training is often required once a CB is hired into a full-time position. This article presents a comprehensive CB training strategy that can be adapted to any collaborative biostatistics group. This strategy follows a roadmap of the biostatistics collaboration process, which is also presented. A TIE approach (Teach the necessary skills, monitor the Implementation of these skills, and Evaluate the proficiency of these skills) was developed to support the adoption of key principles. The training strategy also incorporates a “train the trainer” approach to enable CBs who have successfully completed training to train new staff or faculty.

## Introduction

Science and medicine research initiatives are increasingly interdisciplinary, and the advances in technology demand the ability to acquire, store, analyze, and interpret data. This has led to an increasing need to integrate collaborative biostatisticians within scientific research teams to meet the demand. Many Academic Medical Centers (AMCs) facilitate efficient and reproducible research with centralized collaborative resources that provide access to quantitative experts. There are numerous models and considerations for building and maintaining these groups, including training, evaluating, and establishing realistic expectations for biostatisticians [1–5]. The training and professional development of collaborative biostatisticians (staff and faculty) are necessary to maintain successful and effective biostatistics collaborative programs in AMCs, as well as in institutions focused on clinical or translational science. This article provides a roadmap for the biostatistics collaboration process and proposes specific training strategies to support professional development of clinically integrated collaborative biostatisticians (CBs).

**Fig. 1.**
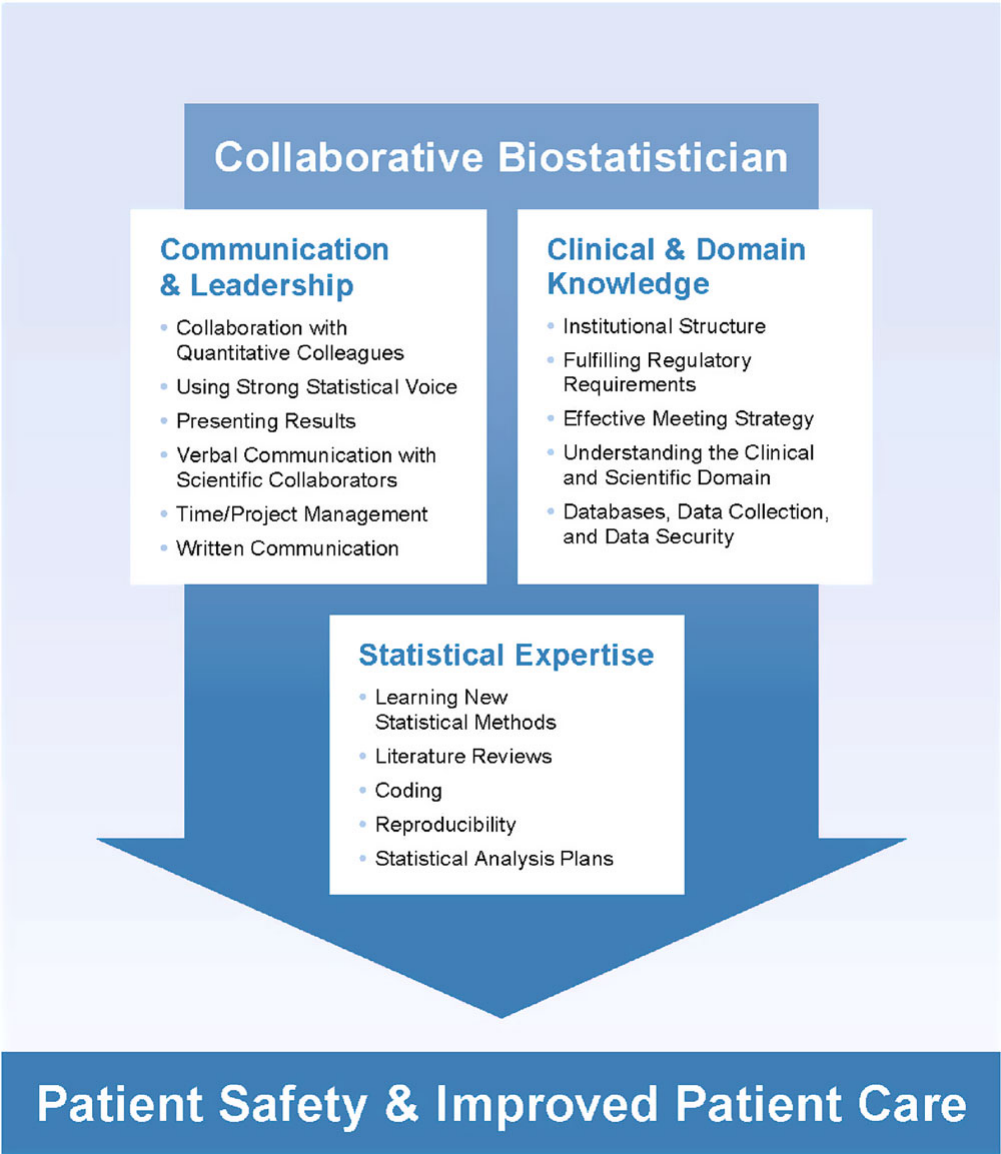
Essential skills of a collaborative biostatistician.

In scientific research, including clinical and translational research, the CB is responsible for ensuring that the design approaches and analytic methods are sound and sufficiently rigorous, so that reasonable inferences and/or accurate predictions can be made. It is imperative that CBs work closely with their clinical and/or translational collaborators as well as with research stakeholders (institution leadership, funding entity or sponsor, etc.) to ensure that results are interpreted appropriately to inform clinical practice and decision-making that will ultimately affect patient care or health outcomes. This requires CBs to provide high-quality analyses, facilitate reproducible research workflows, and communicate statistical results effectively in interdisciplinary collaborative environments. It is important to provide CBs with a clear set of principles from which they can build a solid foundation for implementing sound scientific, business, and regulatory practices. CBs must be able to articulate the purpose and importance of their work to their collaborators and stakeholders. Additionally, CBs must have broad knowledge and understanding of statistical approaches that can be applied in developing, implementing, and revising analytic methods that will enhance the quality of the research. This knowledge will also help facilitate the application of standard and sometimes simpler methods that are pragmatic, thereby increasing both efficiency and interpretability. Equally important are the skills needed to develop robust workflows to ensure that the research goal is achieved in a reproducible manner that upholds the scientific integrity of the project, and that all ethical guidelines for statistical practice are followed [6]. This requires CBs to demonstrate good judgment, effective communication, and methods that will efficiently strengthen scientific rigor. Fig. 1 displays the essential skills needed for success as a CB and provides guidance that should be incorporated in hiring and training practices. The topics and skills presented in Fig. 1 were collected from the current literature [5,7–10] and through anecdotal experience of the leadership of 13 biostatistics units across academia and industry who have vast experience training CBs. The goal of this article is to present and describe comprehensive training methods that can be adapted to any quantitative collaboration group when training staff and faculty CBs, including new or existing units as well as biostatisticians who are embedded within clinical units.

The Joint Task Force for Clinical Trial Competency, a diverse working group tasked with creating a core competency framework for clinical research, has developed a framework that consists of 47 competency statements within 8 competency domains, each described at the “Basic”, “Skilled”, and “Advanced” skill levels [11–14]. This is a comprehensive list for clinical team members; however, it does not reference specific CB competencies or collaboration with CBs. Several articles have previously discussed the need for effective interdisciplinary collaboration and have collectively outlined core competencies needed for CBs to be successful in AMCs [7–10,15]. Begg and Vaughan (2011) reviewed program requirements used to teach interdisciplinary skills at ten top-rated schools and provided a review of related literature. They report that to improve the necessary skills training required for effective interdisciplinary research collaborations, some analytically focused degree programs (biostatistics, epidemiology, etc.) train students to become skilled team scientists. Such programs provide foundational education for CBs. Several articles discuss how these competencies might be integrated into educational curricula and summarize key competencies as: broad and high-level expertise in statistical theory and methods, broad understanding of specific and relevant biomedical areas, and communication and leadership [7,9,10,15]. Previous literature focuses on developing general frameworks, defining competencies, and integrating training into curriculum design. This article specifically focuses on providing additional material concentrated on training CBs to fill gaps in the existing literature. The competencies outlined in the previous literature, along with experience by the authors, were used to develop the proposed collaborative roadmap as well as targeted training strategies for CBs in practice.

Biostatistics and other quantitatively focused degree programs sometimes include training to develop consultation or collaboration skills. However, such training often offers limited exposure to the full collaborative process. Additional training, such as experience in real collaborations and/or working with actual patient data, can be obtained through graduate research assistantships or intern experiences, particularly when in the setting of a high-volume collaborative AMC. Students who complete their degrees in graduate programs that include extensive collaborative training and are then hired into a full-time CB position will often still require additional job-based training and mentoring to equip them with the skills needed to succeed as an effective interdisciplinary collaborator. Additionally, those transitioning into a CB role from a variety of quantitative backgrounds (e.g., theoretical statistics, data science, or epidemiology) or from graduate programs other than biostatistics may need additional training related to specific methods or soft skills to excel as part of an interdisciplinary team.

In some academic institutions, biostatistics faculty take on a collaborative role, and in others, staff are also expected to serve as interdisciplinary collaborators. Spratt et al. (2017) describe the challenges of building career ladders and providing clear expectations for collaborative biostatistics faculty in an AMC [16]. To the best of our knowledge, there is a paucity of literature discussing these topics for research staff (Master’s or PhD level). There are also differences in the structural placement of biostatistics faculty and staff within institutions, with some institutions hiring directly into clinical departments, while others build a centralized core group of biostatisticians who collaborate across multiple clinical disciplines. Biotechnology and pharmaceutical companies also vary widely in the structure of biostatistics units. The proposed training program is sufficiently comprehensive, such that it can be easily modified and incorporated into any of these settings.

**Fig. 2.**
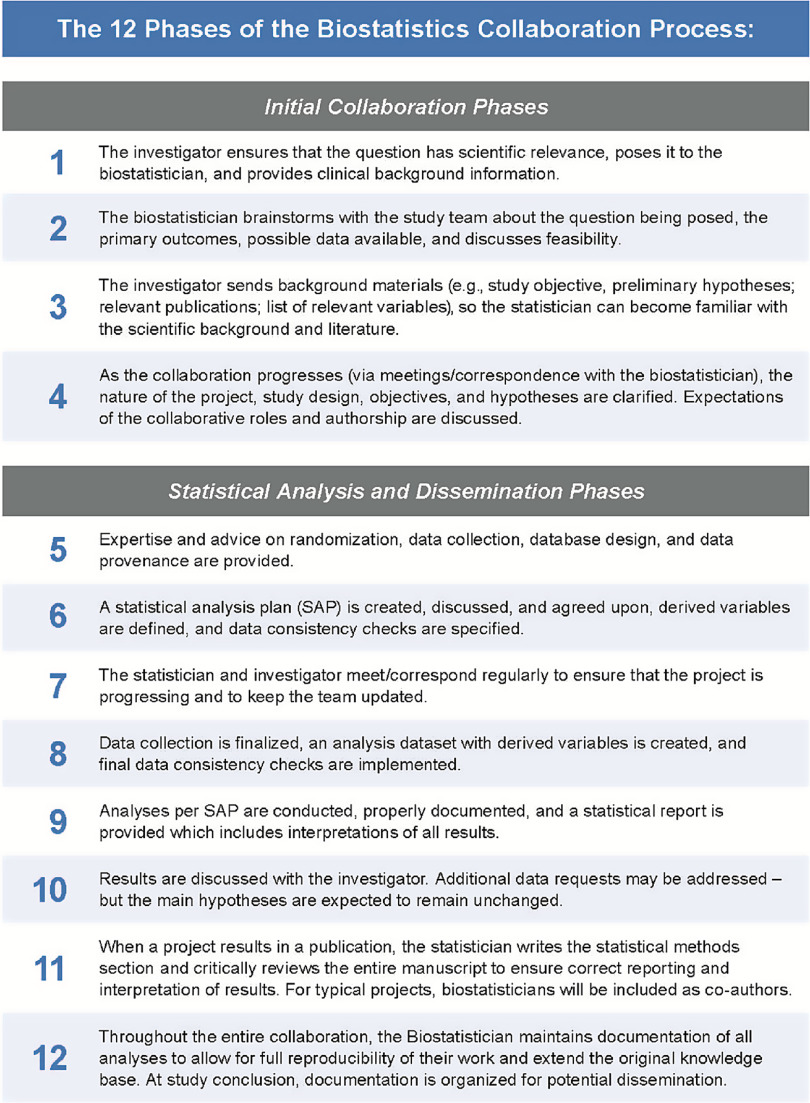
The 12 phases of the biostatistics collaboration process.

The training methods presented in this article were initially developed by the Duke Biostatistics, Epidemiology, and Research Design Core (BERD Core) and have been in use for the last four years to train CBs who have collaborated on more than 700 projects resulting in more than 400 publications and presentations. The training addresses skills that are necessary to navigate through every phase of the research process, from research question development and study design specification through analysis, publication, and/or implementation. To outline the collaborative process, Fig. 2 provides a roadmap that describes the key phases of the biostatistics collaborative process that CBs are trained to navigate. Subsequently, the training methods and collaboration roadmap were agreed upon by leadership of biostatistics units at 13 different institutions and companies. While the material presented here is specifically proposed for biostatisticians, it could be adapted for use with other interdisciplinary research-oriented groups including but not limited to clinical or translational data scientists, biomedical informaticists, and imaging scientists. In this article, first, the collaboration roadmap for biostatistics is proposed; then, a novel training strategy for CBs to navigate this roadmap with tangible examples is presented, and finally, we discuss previous cases of the training methods.

## The Biostatistics Collaboration Roadmap and Training Methods

In each of the 12 phases of the biostatistics collaboration process (Fig. 2), the CB has a key role that requires specific training to support. It is expected that the CB will ensure appropriate completion of most of the phases of the collaborative process, especially when a project manager is not involved. As such, these individuals must be able to take the initiative to move projects forward and meet appropriate deadlines. As a biostatistician gains experience with a specific clinical or scientific research area, they develop expertise regarding common outcomes, data structures, and analytic methods. As this expertise advances, collaborations in this research area become increasingly effective and efficient. Long-term collaborations are strongly encouraged, as they foster the development of strong partnerships and allow important statistical concepts and approaches to become fully integrated into the team’s research endeavors. The roadmap presented in Fig. 2 represents a generalized collaborative process. Research collaborations typically are not linear and may iterate through these steps depending on the project details, funding models, and overall project goals. CBs may join a collaboration after study design has occurred, data collection is complete, or even after a first failed try at publication. While the role of the CB throughout the process will vary by project, it is important for all CBs to understand what their contributions may involve during each phase of the collaboration.

### Initial Collaboration Phases (Phases 1–4)

The first phase of the collaborative process is the initial discussion between the biostatistician and scientific collaborator(s), which optimally should occur while investigators are developing their research questions. Including the CB in this initial step allows them to fully participate in the brainstorming and development of primary and secondary research questions and associated statistical hypotheses. The CB may offer insight into the most statistically appropriate way to frame the question, as well as providing input on methods for data collection or identification of existing datasets that will be the most appropriate. The CB then needs to understand additional clinical/scientific background information, literature, data sources, related outcomes, and limitations. This process should involve ongoing conversations that ensure ample opportunity for the CB to seek clarity when not familiar with a concept. Expectations of the collaborative roles and authorship should be discussed early on within the collaborative process [17].

### Statistical Analysis and Dissemination Phases (Phases 5–12)

The next phase involves outlining appropriate statistical analysis strategies that will eventually evolve into a detailed statistical analysis plan (SAP). The SAP serves to outline the study background, goals (often research hypotheses), data specifications, response and explanatory measures, and all proposed statistical methodologies that will be used to address the research questions. An example template SAP can be found on the Duke BERD Core website [18]. Once the SAP is completed and agreed upon by collaborators, data collection or acquisition of an already existing dataset can commence. The CB may or may not be involved in the day-to-day activities of data collection, but should be in regular communication with those performing this task. Prior to initiating final statistical analyses, the data should be thoroughly cleaned and reviewed, and a “locked” dataset should be created either by or under the direction of the CB. Analysis per the SAP should then be completed using this dataset, results should be summarized by the CB in the form of an analysis report, and statistical inferences from the results should be explained by the CB. In the case of a continuous improvement project, one locked dataset may not be feasible, but datasets used to make decisions at each stage should be stored and documented as appropriate to ensure reproducibility. A formal report should be created that includes all relevant information, and the CB should collaborate on the dissemination of the information (e.g., co-authoring manuscripts). After final dissemination and/or implementation is complete, the CB should package all the work in a way that allows for full reproducibility of their contributions [19–21]. As the CB gains expertise in the specific scientific area through long-term collaborative engagement, this can facilitate efficient navigation through all phases.

### Training for the Biostatistics Collaboration Process

The training strategy, initially developed in the Duke University Biostatistics, Epidemiology, and Research Design (BERD) Core, outlines clear expectations for supervisors who are charged with training junior biostatisticians, and is sufficiently flexible to be adapted for a variety of settings. To ensure biostatisticians are able to effectively navigate the phases of the collaborative process, we propose training that focuses on 16 topic areas. These areas relate to three main core competency areas, as suggested by the current literature [7]: (1) Clinical and Domain Knowledge (broad understanding of specific and relevant biomedical areas, Table 1A); (2) Statistical Expertise (broad and high-level expertise in statistical theory and methods, Table 1B); and (3) Communication and Leadership (including organizational behavior, Table 1C).

**Table 1.** Specific skills essential to TIE process

A) Clinical and Domain Knowledge		
**Databases, Data Collection, and Data Security**	Teach: *What CBs should learn*	1. The data generating mechanism for all relevant data types (EHR, retrospective, prospective, and observational). 2. How to collaborate with database teams. 3. How to interpret and understand of CRFs and DCFs. 4. The specifics of database documentation. 5. How to find and understand documentation about data sources such as white papers. 6. How to conduct a critical review of database builds to ensure all required elements are included and appropriately collected. 7. The data security requirements such as saving data in secure drives and using de-identified data when possible. 8. Institutional or corporate IRB requirements.
	Implement: *What CBs should do*	1. Review resources provided for a thorough understanding of the types of data and follow-up in a meeting with the scientific collaborator to ask questions about the data source. 2. Prepare and shadow senior biostatisticians in meetings with the database team and note the types of questions senior biostatisticians ask. 3. Review sample CRFs and/or other data source information to create a data dictionary with necessary data elements and their specifications. 4. Obtain and interpret database documentation from scientific collaborators. 5. Read the database white papers or internal documentation to understand the features of the database. 6. Review the database build to make sure it collects all data elements needed to complete the study with clear specifications and definitions as specified in the white papers and documentation. 7. Access secure drives for data storage. 8. Read materials on data security and learn about best practices. 9. Review institution or corporate IRB requirements and become familiar with IRB resources and websites, including learning how to verify that they are included on key personnel.
	Evaluate: *What Supervisors should do*	1. Evaluate CBs’ ability to explain the different types of data, and the advantages/disadvantages and limitations of each. 2. Evaluate a CB-led database team meeting on the CB’s ability to discuss all relevant data elements, definitions, and specifications. 3. Evaluate how well CBs can translate CRFs into a data dictionary. 4. Evaluate the following: a. Can CBs describe the data structure of the database? b. Can CBs describe how the database was generated (e.g., sampling scheme, available years, study population, etc.)? c. Can CBs describe the caveats and limitations of using the database? 5. Evaluate the quality of CBs’ description of the database features. 6. Conduct independent review of database build compared to the protocol and evaluate the review conducted by CBs. 7. Review “save locations” of CBs’ work and verify that it is secure. 8. Require CBs to provide a summary of data security and best practices and monitor that CBs are following guidelines. 9. Verify that CBs understand IRB requirements and can confirm that they are listed as key personnel.
**Understanding the Clinical/Scientific Domain**	Teach: *What CBs should learn*	1. A critical understanding and evaluation of clinical/scientific papers. 2. The importance of continuous learning and development. 3. Real-world knowledge of medical practice.
	Implement: *What CBs should do*	1. Summarize the important messages from a sample clinical paper and carefully analyze the statistical analysis section applying the critical appraisal checklist [25]. 2. Attend clinical research seminars to observe how clinicians present research. 3. Shadow scientific collaborators on a clinic visit or watch a medical procedure video to familiarize themselves with the important factors when considering a medical procedure and how they are performed.
	Evaluate: *What Supervisors should do*	1. Evaluate the CBs’ ability to describe the study objectives, study design, statistical methods, and results, and the CBs’ ability to draw conclusions from the paper. 2. Evaluate CBs’ ability to describe the differences in how clinicians and statisticians present results and what can be learned from clinical seminars. 3. Evaluate the CBs’ description of the medical procedure and important factors when considering a medical procedure.
**Fulfilling Regulatory Requirements**	Teach: *What CBs should learn*	1. The relevant regulatory requirements for agencies such as FDA, ICH, NIH, etc., and associated reporting requirements (e.g., ClinicalTrials.gov). 2. Institution-specific guidelines and requirements for clinical research. 3. Standard/good clinical research practice (GLP/GCP). 4. Data protections and privacy requirements.
	Implement: *What CBs should do*	1. Discuss questions that come up when reviewing NIH and FDA guidelines with senior staff. 2. Describe the institutional process and specifics to senior staff and study team members. 3. Complete all required CITI modules including Good Clinical Practice and Responsible Conduct of Research [24]. 4. Implement all requirements as appropriate within their active research projects.
	Evaluate: *What Supervisors should do*	1. Evaluate if CBs are following required regulatory guidelines and appropriately conducting research/analysis in accordance with guidelines. 2. Review and evaluate all regulatory documentation or reports created by CBs to be submitted to NIH, FDA, or internally. 3. Verify that CBs receive certification of completion of CITI Modules and other standard clinical research training.
**Institutional Structure**	Teach: *What CBs should learn*	1. The structure and requirements of medical education in the U.S. (preclinical and clinical training in the medical school, clerkship, residency, fellowship, faculty). 2. The structure of the academic medical institution or collaborative company. 3. The types of grants and funding opportunities offered by NIH, PCORI, etc., and the associated application documents and review processes.
	Implement: *What CBs should do*	1. Summarize structure and requirements of medical education in the USA. 2. Review the academic medical institution website or company organizational chart to understand the structure of the institution (e.g., departments, divisions) and the training or education programs of the institution (e.g., residency and fellowship programs) or company (e.g., internship programs). 3. Review the NIH Grants & Funding website to understand types of funding opportunities (research/training/small business grants, etc.) and types of applications (new, renewal, etc.). 4. Understand grant application review process and common acronyms (e.g., R01, RFA, FOA, etc.).
	Evaluate: *What Supervisors should do*	1. Evaluate the CBs’ ability to describe the structure and requirements of medical education in the USA. 2. Evaluate the CBs’ ability to explain the structure of the institution and the differences in medical students, residents, and fellows. 3. Review the CBs’ ability to explain different funding mechanisms, the grant application review process, and the scoring system. 4. Evaluate the accuracy of the grant application review process and the definitions of common acronyms.
B) Statistical Expertise		
**Statistical Analysis Plans**	Teach: *What CBs should learn*	1. The necessity of statistical analysis plans. 2. The content of the background, hypotheses, and research questions, and the ability to write appropriate statistical methods to answer clinical/research questions. 3. Process for creating a of full statistical analysis plans for variety of studies.
	Implement: *What CBs should do*	1. Critically review sample SAPs. 2. Leverage online trainings to understand the importance of SAPs and practice explaining this to scientific collaborators. 3. Write a draft SAP for a current project using templates provided by supervisor.
	Evaluate: *What Supervisors should do*	1. Evaluate gaps and strengths in the CBs’ draft SAPs. 2. Evaluate CBs’ ability to explain the necessity of the SAP. 3. Evaluate the following: a. How well the scientific problem is explained. b. Are there additional questions that need to be clarified with the scientific collaborator? c. Does the statistical methods section match the questions being asked? d. Are the methods appropriate for the data and research question?
**Reproducibility**	Teach: *What CBs should learn*	1. The importance of reproducibility in medical research. 2. The ability to use source code management software such as git. 3. Ways in which CBs ensure reproducibility in their work, including rmarkdown/sweave (R) and batch submit/ODS output (SAS), and version control. 4. The need for standardized processes and documentation, including SOPs and MOOs. 5. The importance of professional ethics in statistical practice.
	Implement: *What CBs should do*	1. Read reference materials that explain reproducibility in research and discuss with supervisor/manager. 2. Learn basic functions of git and implement it for projects. 3. Incorporate rmarkdown/sweave (R) or batch submit/ODS output (SAS) into projects as necessary. 4. Review the Ethical Guidelines for Statistical Practice [6].
	Evaluate: *What Supervisors should do*	1. Ask CBs to describe reproducibility and professional ethics and why they are important, and evaluate their understanding. 2. Review the workflow and implementation of git by the CBs and evaluate their understanding of basic git functions and implementation. 3. Audit and review CBs’ project folders to ensure the use of rmarkdown/sweave or batch submit/ODS output.
**Coding**	Teach: *What CBs should learn*	1. Good programming practices [26]. 2. Knowledge of the key elements of good analysis code, e.g., appropriate header, comments, and sectioning. 3. Knowledge of general statistical good programming practices that involve modular programming, results output, minimizing copying and pasting output.
	Implement: *What CBs should do*	1. Review sample projects that follow good programming practices and provide comments and documents specific elements they deem positive or negative. 2. Create header template that can be used for all programming scripts. 3. Implement good programming practices in projects.
	Evaluate: *What Supervisors should do*	1. Evaluate comments and listing of good/bad elements by CBs and provide feedback on any apparent gaps. 2. Evaluate header template created by CBs and provide feedback. 3. Review CBs’ project folders and code for good programming practices.
**Literature Reviews**	Teach: *What CBs should learn*	1. How to review literature for current statistical methods. 2. How to use literature websites such as PubMed and Google Scholar in addition to librarians and resources available in academic libraries. 3. Familiarity with software programs such as Endnote Mendeley, Zotero, etc.
	Implement: *What CBs should do*	1. Practice conducting literature reviews based on hypothetical research questions. 2. Identify papers using similar methods compared with an assigned publication that implements a complex methodology. 3. Review relevant software programs and reference materials, and work through an assigned test project.
	Evaluate: *What Supervisors should do*	1. Evaluate if the literature review is comprehensive given the research question. 2. Review publications with similar methodology and evaluate if CBs can now describe the methodology in more detail after conducting literature review. 3. Assign CBs tasks that require use of relevant software programs and evaluate performance.
**Learning New Statistical Methods**	Teach: *What CBs should learn*	1. Self-instruction and implementation of new statistical methods as required by ongoing collaborations. 2. The critical review of new methodology to determine appropriateness in ongoing collaborations.
	Implement: *What CBs should do*	1. Practice implementing new methodology based on review of papers and reading materials. a. If available, provide sample code. b. If necessary, review theory behind methodology. 2. Use literature review methodology above to identify potentially relevant papers and methods. a. Read and take notes on all identified papers/methods.
	Evaluate: *What Supervisors should do*	1. Meet with CBs to discuss questions about new methodology and review analysis to confirm accuracy. 2. Meet with CBs to discuss all identified methodologies. 3. Evaluate CBs’ ability to describe the pros and cons of each method (including but not limited to assumptions, efficiency, generalizability, etc.).

C) Communication and Leadership		
**Written Communication**	Teach: *What CBs should learn*	1. The importance of consistently acknowledging the receipt of scientific collaborator emails within a pre-specified time frame (e.g., within one business day), and, if needed, communicating when one will be able to dedicate time to any requests. 2. How to construct succinct and clear emails to scientific collaborators and other methodologists. 3. When to send an email vs. initiating a verbal conversation.
	Implement: *What CBs should do*	1. Respond to all emails within a pre-specified time frame and copies supervisor on these emails. 2. Develop an email signature for use in all email correspondence. 3. Use templates and other resources provided (dos and don’ts, example emails, etc.) to word their emails professionally. 4. Articulate decision process behind verbal vs. email communication.
	Evaluate: *What Supervisors should do*	1. Audit emails to see if CBs are: a. Sending and responding to emails professionally. b. Responding to all emails within the pre-specified time frame. c. At least initially, using templates or similar wording to send professional emails. d. Including professional email signature on all correspondence. 2. Provide CBs with an example email for them to respond to and evaluate responses crafted by CBs. 3. Evaluate if any emails should have been replaced with verbal conversations. Role-play/discuss to understand situations that would require verbal conversations.
**Time/Project Management**	Teach: *What CBs should learn*	1. How, why, and when to use a task manager. 2. How to block calendar time for project work. 3. How to plan proposed timelines in the SAP, and how to send reminder emails to supervisors/collaborators about pending or changing timelines. 4. How to accurately project timelines and expected lead time for requests from scientific collaborators. 5. How to request that a deadline be reconsidered or postponed. 6. How to elicit timeline requirements from scientific collaborators and ensure clear communication of deliverables and timelines with careful delineation of assumptions and potential risks (i.e., scope clarity). 7. How to work with collaborators to establish project priorities when simultaneous projects exist. 8. How to allocate appropriate time and effort when supporting multiple teams, perhaps establishing mutually agreed-upon, regularly recurring blocks of time for each team.
	Implement: *What CBs should do*	1. Use calendar and task manager software, with examples and guidance provided by supervisor. 2. Send task lists to supervisor each week, with time estimates for each task, and assessment of time actually spent on tasks the previous week. (Continue until this skill is mastered.) 3. Create timelines and steps for completing projects with ideal proposed deadlines in SAP. If deadlines are shifted, communicate this clearly via written communications. 4. Review examples of emails and other communication provided by supervisor to discuss setting deadlines and re-evaluating timelines. 5. Shadow supervisor to see how timelines are mapped out with scientific collaborators. Supervisor provides guidance on enforcing required lead time for completion of tasks requested by scientific collaborators. 6. Include timelines on SAPs and clearly communicate this in emails. 7. Work with supervisor to map out timelines for projects as appropriate. 8. When simultaneous projects exist within a collaboration, note the mutually agreed upon priority ordering of projects. 9. When supporting multiple teams, work with project supervisor and team leads to agree to a regular schedule for each team’s work.
	Evaluate: *What Supervisors should do*	1. Audit calendar and task lists to confirm that CBs are using them, and ask for feedback on how it’s going. 2. Provide feedback on task lists, specifically noting where time estimates were way off and discovering why the estimates were off (e.g., tangents). 3. Evaluate CBs’ understanding of setting timelines and priorities by conducting mock discussions involving multiple projects with reasonable and unreasonable timelines, providing feedback to CBs based on how they navigate the discussion. 4. Evaluate CBs’ ability to provide required lead time when prompted based on the type of activity. 5. Evaluate the previously proposed timelines and determine if CBs were able to meet them. 6. Discuss what is causing delay in deliverables and ways to properly address the delays. 7. Verify that CBs’ time spent with different teams respects effort allocation agreements.
**Effective Meeting Strategy**	Teach: *What CBs should learn*	1. The structure and flow of successful meetings. 2. The ability to critically interview supervisors and scientific collaborators. 3. The different responsibilities of junior staff, senior staff, and faculty on each project.
	Implement: *What CBs should do*	1. Shadow experienced staff in both initial and follow-up meetings with scientific collaborators. 2. Take notes and write questions during shadowing meetings for follow-up and further discussion. 3. Participate in mock experiences with supervisors and/or managers to practice investigatory skills. 4. Discuss differences in job responsibilities and expectations, including level of independence between junior and senior CBs in meetings with scientific collaborators (i.e., when is it appropriate for CBs to meet with scientific collaborators without including supervisor?).
	Evaluate: *What Supervisors should do*	1. Evaluate ability of CBs to ask questions directly to scientific collaborators in meetings. 2. Evaluate notes and written questions for comprehension and detail to discuss/determine what should or should not have been asked. 3. Evaluate and provide feedback on appropriateness and thoroughness of questions during mock experiences. 4. Evaluate CBs’ level of independence during meetings and discuss whether this is appropriate for the job level. 5. Deploy surveys and qualitative feedback mechanisms for scientific collaborators to provide feedback regarding CBs’ communication process in meetings.

**Verbal communication with Scientific Collaborators**	Teach: *What CBs should learn*	1. How to identify the research question the scientific collaborator is truly interested in answering. 2. How to determine the data that was/will be collected. 3. How to focus first on the scientific question rather than the statistical methods, and recognize that the methods should be determined after fully understanding the data and the research question. 4. How to develop active listening skills during collaborative meetings. 5. How to provide to scientific collaborators a regular summary of the progress made on ongoing projects.
	Implement: *What CBs should do*	1. Shadow other biostatisticians in meetings. 2. Practice what they have learned in meetings with scientific collaborators by formulating study questions and describing data collection processes and mechanisms. 3. Participate in role-playing with supervisor or other staff CBs to practice conducting effective and efficient discussions with the scientific collaborator. 4. Send succinct weekly emails highlighting project progress, perhaps on Friday, to supervisor and collaborative teams. 5. Practice soft skills such as explaining using storytelling or analogies [22,23].
	Evaluate: *What Supervisors should do*	1. Observe CBs’ mannerisms and participation in meetings with scientific collaborators and provide feedback. 2. Evaluate CBs’ ability to actively participate in meetings by reviewing the topics discussed and the quality of discussion with regard to effectiveness and efficiency. 3. Employ the teach-back method of asking CBs to repeat what they heard/learned in a meeting, in their own words; provide feedback. 4. Read status updates sent from the CBs to research teams, providing feedback if needed.
**Presenting Results**	Teach: *What CBs should learn*	1. How to communicate results to a non-statistical or non-technical audience, both verbally and in writing. 2. How to create comprehensive work products and how to present results verbally and in writing in a clear and concise way.
	Implement: *What CBs should do*	1. Shadow senior staff during presentation/results meetings with scientific collaborators. Note questions to be reviewed with supervisor. 2. Review analysis results (i.e., tables and figures) and practice writing summaries for report and/or manuscript. 3. Practice presenting results to supervisor and other biostatisticians with predetermined common questions that a non-statistician might ask. 4. Prepare instructional presentations for colleagues and/or scientific collaborators aimed at teaching the methods that were used and conveying the interpretations of the results.
	Evaluate: *What Supervisors should do*	1. Review and evaluate questions CBs formulated from presentation meetings. 2. Review and evaluate report write-ups with CBs for accuracy and writing skills. Make edits as necessary, but explain why edits are being made. 3. Provide feedback from practice sessions about strengths and areas for improvement, addressing how those improvements can be made. 4. Conduct practice sessions with CBs for instructional presentations and provide feedback. Provide one-on-one formal feedback after the instructional presentation is given to address specific areas for improvement.
**Using Strong Statistical Voice**	Teach: *What CBs should learn*	1. How to identify attainable research goals. 2. How to advocate assertively but respectfully one’s position to a scientific collaborator when a particular application of a statistical method is not feasible or additional considerations need to be made. 3. The intricacies of the leadership structure and how to identify senior biostatisticians and/or faculty who can serve as support to CBs.
	Implement: *What CBs should do*	1. Practice translating research statements into testable hypotheses. Provide example research statements with varying levels of specificity. 2. Shadow initial meetings and observe how senior biostatisticians ask pointed questions to draw out research goals and document any observations or questions from these meetings. 3. Role-play to practice advocating scientific views to scientific collaborators.
	Evaluate: *What Supervisors should do*	1. Review and evaluate research statements and hypotheses developed by CBs. 2. Review questions and notes from shadow meetings. Explain why certain topics were discussed and what each was intended to elicit from the scientific collaborator. 3. Provide feedback on role-play and guide CBs to formulate arguments based on statistical validity; suggest alternative analyses or approaches as necessary.
**Collaboration with Quantitative Colleagues**	Teach: *What CBs should learn*	1. How to identify when to seek guidance from another quantitative scientist or mentor. 2. How to solicit feedback. 3. How to outline timelines for mentors and collaborators.
	Implement: *What CBs should do*	1. Work on a project that will require additional expertise. 2. Write summary emails after every meeting that clearly outline what feedback is needed. 3. Propose timelines for projects that are appropriate and specifically outline when feedback will be needed from supervisors and collaborators within the proposed timeline.
	Evaluate: *What Supervisors should do*	1. Evaluate if the CB sought appropriate expertise or if the supervisor needed to request the expertise. 2. Ensure that CBs copy the supervisor on the summary emails and evaluate how CBs ask for feedback. Follow up and evaluate if the feedback was received and if it was appropriate, and discuss with CBs what went right and or wrong with the interactions. 3. Evaluate if the timelines were met and if CBs are over- or underestimating timelines so they can identify their personal tendencies and adjust.

Abbreviations: CB = Collaborative Biostatistician; CITI = Collaborative Institutional Training Program; CRF = Case Report Form; DCF = Data Collection Form; EHR = Electronic Health Record; FDA = Food and Drug Administration; FOA = Funding Opportunity Announcement; GCP = Good Clinical Practice; GLP = Good Laboratory Practice; ICH = International Council for Harmonisation; IRB = Institutional Review Board; MOO = Manual of Operations; NIH = National Institutes of Health; ODS = Output Delivery System; PCORI = Patient-Centered Outcomes Research Institute; RFA = Request for Application; SAP = Statistical Analysis Plan; SOP = Standard Operating Procedure.

Clinical and domain knowledge includes topics that relate to the responsible conduct of research, knowledge, and understanding of institutional structure, as well as understanding of the clinical/scientific domain.

Statistical expertise refers to the skills needed to implement statistical approaches, develop statistical analysis plans, implement reproducible coding practices, learn new statistical methods as needed, and conduct relevant literature reviews.

Communication and Leadership refers to effective written and verbal communication skills, leading effective collaborative meetings, and time/project management. The importance of developing soft skills (e.g., communication, interpersonal skills, etc.) is also addressed in this section [22,23].

Table 1A–C is organized using these three broad areas, with sub-topics, to allow for training of specific skills. These tables describe the approaches that have been developed to Teach the necessary skills, monitor the Implementation of these skills, and Evaluate the proficiency of these skills (TIE). During the “Teach” stage of training, key learning objectives are formally outlined, and learning strategies are provided by the CB’s supervisor. The “Implementation” stage lists specific tasks that the CB is expected to complete in order to reinforce learning of the topics outlined in the “Teach” stage. In the essential “Evaluation” stage, the CB receives critical feedback and opportunities to identify knowledge gaps that can be addressed through iterative exploration of the TIE process to improve skills and enhance knowledge. Table 1A–C presents the TIE process for each of the three main competencies that a CB must master (see Fig. 1).

At Duke, we have implemented TIE using “train the trainer” and “near-peer” models. The supervisor trains one person and mentors the trainee until they are ready to move into a supervisor role. Their ability to supervise is evaluated using the TIE methodology by leadership in the unit. This model repeats as junior CBs mature, and additional research staff and faculty are hired. The goal is that all trained CBs, within any role and in any collaborative clinical or translational science setting, could train other CBs who are junior to them. Evaluations can be conducted informally during regular management and/or as part of a formal annual evaluation process. At Duke, evaluations are conducted via annual online surveys in which biostatistics supervisors and collaborators rate the staff member’s ability in each competency category (on a Likert scale). Regular evaluations of each skill in Table 1 are conducted by the staff member’s manager. We present examples in the next section to provide specific methods that can be used for teaching, implementing, and evaluating specific skills.

### Example of TIE in Clinical and Research Domain – Fulfilling Regulatory Requirements

All CBs must be sufficiently trained in regulatory requirements for research. Failure to follow regulations can result in termination of IRB approval, withdrawal/restriction of research funding, and fines. CBs may need to be knowledgeable about NIH and FDA guidelines and regulations for clinical research, institution-specific guidelines and regulations for scientific research, and standard/good scientific research practice.

#### Teach

The “Teach” stage may involve the CB viewing online learning modules, attending in-person trainings, and formally reviewing NIH, FDA, or other relevant guidelines with structured discussion led by senior/supervising biostatisticians.

#### Implement

The CB may be required to implement institutional processes and complete all in-person and online institutional and commercial training modules (e.g., Collaborative Institutional Training Initiative Program [24]). Additionally, the CB could be tasked with explaining processes and specifics to other CBs and the study team to demonstrate mastery of the topics. In this stage, CBs would work to implement all requirements as appropriate within their active research projects and discuss questions with their supervisors.

#### Evaluate

Supervising biostatisticians will conduct a formal review to confirm that the CB has acquired the skills necessary to follow required regulatory guidelines and perform research and analyses in accordance with guidelines. These skills will be evaluated through detailed review of their work and verification that all required regulatory elements are in place (e.g., listing as key personnel on protocols). Supervisors will also evaluate the CB by critically reviewing all regulatory materials created by the CB to be submitted internally, or to the NIH or FDA. The final step in the Evaluation will be the CB’s successful completion of online and in-person training (indicated by receiving confirmation or certification of completion of post-training tests).

### Example of TIE in Statistical Expertise – Statistical Analysis Plans (SAPs)

Creation of the SAP requires understanding of the background, research questions, and hypotheses, as well as the data structure and statistical methodology that will be used to answer the research question. CBs must effectively communicate with the study team and review relevant materials to fully understand the scope and goals of a project in order to draft the SAP. Drafting of the SAP and acquiring final approval from the scientific collaborator ensures that both parties agree on the project specifics (i.e., analytic approaches, outcomes, data, etc.) and expectations of each role within the collaborative setting [18].

#### Teach

The CB will work to understand why SAPs are necessary and the specific elements that should be included. They will also learn the process for creating SAPs for a variety of study types. To teach this skill, the CB can review SAPs from the supervisor’s previous projects and discuss potential issues that could have occurred if all key components were not included. A template SAP can be provided, so the CB can understand all key components. The supervisor can also task the CB with writing the first draft of an SAP, which can then be compared to the draft written by the supervisor, followed by discussion of differences. This technique will help the CB think about why they missed certain key points and help them in drafting future SAPs.

#### Implement

The CB critically reviews sample and example SAPs, participates in online training, and writes draft SAPs using templates provided by the supervisor. The CB may also be asked to practice explaining what SAPs are and why they are important to peers, the supervisor, or scientific collaborators (with guidance and oversight from supervisor). They will also be asked to draft SAPs for projects they are collaborating on, with supervision.

#### Evaluate

The supervisor will review the draft SAPs created by the CB, identify gaps and strengths, and provide constructive criticism verbally and in writing. The supervisor will provide the CB with a list of additional questions that should be clarified by the scientific collaborator. Over time, the supervisor can track how many questions the CB missed in order to evaluate growth. Evaluation of competency with this skill could also entail a reduction in the number of edits needed by the supervisor.

### Example of TIE in Communication and Leadership – Using Strong Statistical Voice

A strong statistical voice in this context is considered to be the ability to confidently and accurately advocate for appropriate quantitative methodology and resulting interpretations. CBs must be able to actively listen, absorb, and convey their opinions during all aspects of collaborative research, from study design and hypothesis generation to results interpretation and dissemination. CBs must also serve as advocates for the use of appropriate methodology and interpretation, something that can be challenging when scientific collaborators are unfamiliar with statistical methods or do not obtain the results they were hoping to see [22]. Using a strong statistical voice is an important skill that can affect which methods are used and how results are interpreted and disseminated. Therefore, a strong statistical voice is vital in study planning, especially when selecting outcomes of interest, designing the data collection approach, and conducting power and sample size calculations. If the CB does not effectively communicate which analytic approaches are most appropriate, studies may suffer from poor design and statistical errors or fail to achieve desired power to detect results. Similarly, if a CB does not ask the right questions or communicate effectively with a scientific collaborator to identify critical variables that should be collected and analyzed, the results may not accurately address the research questions of interest.

#### Teach

Skills to “Teach” include the importance of gaining an in-depth understanding of the research questions and critical evaluation of introduction and background material to ensure sufficient understanding of the motivation for the research project. Additionally, when appropriate, assertive advocacy of one’s position regarding appropriate methodology or lack of appropriate data to properly address a research question is important. This can include the ability to balance realistic modifications to statistical methodology proposed by collaborators while ensuring rigor and reproducibility of results. A clear and detailed understanding about leadership structure and how to identify senior-level biostatisticians and/or faculty to serve as support is needed.

#### Implement

These skills can be “Implemented” by providing examples of research statements to CBs and requiring them to translate these statements to research questions and testable hypotheses. Additionally, CBs can shadow collaborative meetings and be required to document how senior biostatisticians ask targeted questions and advise on appropriate analytical approaches.

Another approach to implement this skill involves having CBs role-play with their supervisor, so they can practice advocating for appropriate methodologies and research considerations in different types of challenging research project settings.

#### Evaluate

Supervising biostatisticians can then “Evaluate” these skills by observing how the CB communicates thoughts and concerns in a team meeting and through email communications. The supervisor can then provide feedback and discuss additional questions or methods that the CB should have considered. Peer-to-peer role-playing may also be an effective tool to incorporate, as it would allow the CB to focus on growth and practice without the added pressure of evaluation. Evaluation for the role-playing scenario can be targeted to evaluate specific communication skills. For example, the supervisor can play the role of the scientific collaborator who proposes the use of inappropriate methodology to answer the research question, stemming from a lack of understanding of the assumptions of the methodology. The supervisor can then evaluate how well the CB is able to engage in a conversation about why the assumptions would be violated and how effectively the CB can communicate with the “collaborator” and successfully suggest more appropriate methodology. Evaluation should also include providing critical feedback aimed at helping the CB develop reasoned explanations based on statistical validity and alternative approaches as needed.

## Discussion

Given the tremendous impact research has in affecting health care decision making and health outcomes, the development of a highly skilled collaborative workforce of biostatisticians is a critical priority. CBs are becoming increasingly essential to clinical translational science, and it is imperative that CBs are trained to be mindful of their important roles, as they collaboratively design studies, analyze data, and interpret results to help collaborators disseminate study findings. CBs must help assure that data are generated, recorded, and transferred using processes and systems that are secure, efficient, and reproducible. CBs must also work as integral team members to help others (colleagues, regulators, providers/investigators, etc.) draw appropriate statistical inference. To do this, CBs must have broad methodological knowledge and the ability to consider different methods for the design and analysis of studies. If there is any conflict regarding the appropriate statistical methods to be used, the CB should be able to effectively communicate the issues to their collaborators, colleagues, and supervisors to advocate for the correct methods. If errors occur or counterintuitive results are found, the CB must have the skillset to investigate the errors in a timely fashion. Errors should be corrected, noted (as appropriate), and shared to create an iterative learning cycle of unbiased and clear interpretation. CBs should also know when to request independent review to further validate findings. It is not enough that only intent is unbiased, but the CB must work with the scientific collaborator to ensure that objective interpretations are disseminated. Adequate training of CBs to do this has the potential to directly impact the integrity of the research by facilitating more effective collaborations. The methods we present aim to address each of these important challenges in training strong CBs.

The collaborative process and training methods presented here are broad and inclusive, so that they can be adapted to a variety of collaborative situations. In long-term collaborations, all methods may apply, while in short-term or one-off collaborations, selective and relevant parts of the proposed material can be utilized. A CB who is trained in the entire process should be able to identify and apply appropriate steps of the collaborative process.

It is imperative that CBs understand the steps required for project completion and all expectations of their role. For institutions to retain CBs who have developed strong competencies, there must be sufficient time dedicated to implementing effective training methods and providing career development opportunities. If training and opportunity for advancement are not available, the institution risks losing strong researchers. The TIE strategies presented in this article have been used to train more than 22 BERD Core staff and have been extended to train 54 Biostatistics Core Training and Internship Program student interns who have collectively worked on more than 40 collaborative teams across the School of Medicine at Duke University. Initial efforts to expand this approach appear promising and could be adopted widely by the clinical and translational science community. Future work will focus on the implementation and evaluation of these training strategies in order to work toward the development of new strategies.
